# Bioinformatics: indispensable, yet hidden in plain sight?

**DOI:** 10.1186/s12859-017-1730-9

**Published:** 2017-06-21

**Authors:** Andrew Bartlett, Bart Penders, Jamie Lewis

**Affiliations:** 1 Department of Sociological Studies, University of Sheffield, Elmfield, Northumberland Road, Sheffield, S10 2TU UK; 2 Department of Health, Ethics & Society, Care and Public Health Research Institute (Caphri), Maastricht University, PO Box 616, Maastricht, 6200MD the Netherlands; 3 School of Social Sciences, Cardiff University, Glamorgan Building, King Edward VII Avenue, Cardiff, CF10 3WT UK

**Keywords:** Sociology, Career, Interdisciplinarity, History, Expertise, Credit, Reward

## Abstract

**Background:**

Bioinformatics has multitudinous identities, organisational alignments and disciplinary links. This variety allows bioinformaticians and bioinformatic work to contribute to much (if not most) of life science research in profound ways. The multitude of bioinformatic work also translates into a multitude of credit-distribution arrangements, apparently dismissing that work.

**Results:**

We report on the epistemic and social arrangements that characterise the relationship between bioinformatics and life science. We describe, in sociological terms, the character, power and future of bioinformatic work. The character of bioinformatic work is such that its cultural, institutional and technical structures allow for it to be black-boxed easily. The result is that bioinformatic expertise and contributions travel easily and quickly, yet remain largely uncredited. The power of bioinformatic work is shaped by its dependency on life science work, which combined with the black-boxed character of bioinformatic expertise further contributes to situating bioinformatics on the periphery of the life sciences. Finally, the imagined futures of bioinformatic work suggest that bioinformatics will become ever more indispensable without necessarily becoming more visible, forcing bioinformaticians into difficult professional and career choices.

**Conclusions:**

Bioinformatic expertise and labour is epistemically central but often institutionally peripheral. In part, this is a result of the ways in which the character, power distribution and potential futures of bioinformatics are constituted. However, alternative paths can be imagined.

## Background

Richard Feynman is quoted as having said that: the “philosophy of science is as useful to scientists as ornithology is to birds.” Quite probably, many scientists think that something similar is true of the sociology of science. In this commentary, which draws on our previously published research in sociology of science journals, we suggest some ways in which the sociology of the life sciences, and of bioinformatics in particular, *can* be useful to scientists. In particular, we will show that bioinformatic work is operating in a social, institutional, and cultural context that presents obstacles to it receiving due credit despite its increasing importance. Is this the result of the struggles of a discipline coming of age, or is this the early history of a field of inquiry that will be assimilated into big biology? Our ‘ornithology’ therefore is not intended to teach birds to fly, they already know how to do that, but by providing them with a description of their ecology and environment, it is intended to help them choose a destination for their flight.

‘Bioinformatics’ is many things, and this multiplicity isn’t limited to its multi-disciplinarity. For example, it is a field of study, a body of knowledge, a collection of tools and methodologies, a service, a community of journals such as this one, a collection of conferences, and departments, and, importantly, a form of work. As work, it takes effort and skill, brings satisfaction and frustration, often in equal measure, and produces a product. And, like every type of work, it exists entangled in a web of other types of work, a web which includes not only ‘wet’ laboratory work, but also managerial work, accounting work, the ‘soft’ work of social and emotional labour, etc. Now, more than a decade after the completion of the Human Genome Project, bioinformatic work has become an integral part of the web of work in the life sciences – so much so that these threads cannot be entirely picked apart and unravelled. The development and institutionalisation of bioinformatics though has provided us with an opportunity to explore science and scientific practice, and to critically examine bioinformatics’ and bioinformaticians’ roles in the life sciences. Science itself is about producing knowledge, but the day-to-day work of science is also about securing resources, crafting collaborations, earning credit, building reputations, as well as negotiating what it is that counts as ‘important’, ‘relevant’, ‘significant’, or even ‘interesting’. Science, from this point of view, is inextricably bound into the institutional and organisational arrangements that shape and influence the work being done and the people doing the work, as well as the distribution of power between scientists, disciplines, and institutions [1].

## Methods

Over the last decade, we have studied the bioinformatics community, the work they perform and the social, political, and epistemological context that shapes that work. We have used a mixed-methods approach combining qualitative and quantitative data collection strategies. Qualitative data includes, amongst many other things, content-analysis of life science and bioinformatic papers, observational and ethnographic fieldwork at conferences and meetings and interviews with close to 100 bioinformaticians and other scientists working in or with bioinformatics. In addition to this, we also conducted a survey of over 300 UK bioinformaticians which produced both qualitative and quantitative data. From this data, we have produced several papers [2, 3–5, 6]. From these papers, we have distilled a number of key observations on the state of bioinformatics at an epistemic and political level, with consequences for the type, volume and content of work that is being performed and produced, which we present below.

## Results and Discussion

### The character of bioinformatic work

Bioinformatics finds its roots in the conceptualisation of biology as data [7]. While biology and heredity being a matter for calculation has a long history, with the likes of Mendel and Galton quantifying heritable traits well over a century ago, it is only when life scientists started to conceptualise hereditary material as a source of *information*, as opposed to solely *matter*, that calculation and data processing became epistemically central. These origins help explain the dual epistemic roots of the bioinformatics community: skill, expertise and ways of seeing and questioning was drawn into the community from biology, while increasingly information engineers, computer scientists and mathematicians were required and grew fascinated by biology as its datasets grew ever bigger and more complex. The logic of understanding genetic information as information made the incorporation of the expertise of these disciplines appear a ‘natural’ step in the development of the life sciences. Bioinformatics has thus become a central pillar of life science, omnipresent and indispensable, and yet it often blends into the background [8]. Alongside their methodologies, skills and expertise, biologists and computer scientists have also brought their respective research cultures – their values and priorities – into bioinformatics, creating a hybrid inter-discipline and a hybrid culture [3]. This means that not only are there cultural as well as intellectual boundaries between biologists, computer scientists, and bioinformaticians, but there are also points of friction and tension within the broad, heterogeneous field of bioinformatics itself [4, 5], as well as in articulations of how to educate its new members [9, 10].

In the context of the evaluation and audit cultures that are pervasive in contemporary science [11], this hybrid identity creates practical problems for bioinformatic work. What is valued matters and shapes the work produced. If a scientific community makes judgements of academic quality based on authorship of scientific papers and the impact factor of journals, the development of new algorithms and bioinformatic tools will be a devalued, dispreferred activity for academic life scientists. It is not surprising, then, that those we have spoken to have reported that many view bioinformatics as a ‘service’, rather than as a scientific field in its own right. In some cases, the development of tools that are used by life scientists renders the intellectual contribution of bioinformaticians invisible, hidden in the ‘black box’ [4]. As a consequence, despite bioinformatics being central to the current life science landscape, it is often institutionally peripheral, less an academic accomplishment, and more a ubiquitous tool required to do post-genomic science [3].

### The power of bioinformatic work

A clear disciplinary identity is accompanied by stability, a defined institutional role and an historical narrative, which provides justification of that role. Such an identity is part of what grants legitimacy and power to, for instance, well-established biomedical fields. Those within the discipline get to set (and police) the boundaries of that discipline, determine what is to be valued, and how best to produce knowledge. But we live at a time when ‘inter-disciplinarity’ is a buzz-word, promoted by a rhetoric that positions many medical and social problems outside – or between – the boundaries of academic disciplines, and proposes new structures that will fill these spaces and render these complex problems tractable. However, when we look at actually existing research practices, we find that, for many, interdisciplinary work is risky. It falls outside of established power structures, it does not fit evaluation models built for disciplinary scientific work [8] and, related to these facts, it is does not generate the same degree of respect from both peers and public, partly because the lack of a decades-long track record of accomplishments.

Furthermore, while bioinformatic work is central to the life science, it is also highly *dependent* on it. It is laboratory work – the ‘wet-lab’ – that first translates the matter of life into data (producing ‘primary inscriptions’), after which bioinformaticians, working in the ‘dry-lab’, carry out further transformations (producing ‘secondary inscriptions’) [4]. How credit should be distributed between those producing the primary inscriptions and those producing the secondary inscriptions is unclear and cultures of credit distribution are still developing. The same goes for the question of who are to be the legitimate interpreters of these inscriptions. Should it be biologists, for example, or should it be bioinformaticians? We have found the case that those performing (or at least those *heading* the laboratories performing) the work of primary inscriptions have often laid claim on the prestige of first authorship (and often last too), with (much) less prestige afforded to those involved in the work of producing secondary inscriptions [3, 6]. This is a process of negotiation, even if the terms are never explicitly debated, and the result is testimony to the lack of power of bioinformatics in these negotiations. The life sciences are still the domain of biologists. Indeed, our research has found that those doing bioinformatic work feel taken for granted, overlooked, or worse: the legitimacy of their entire research programmes (in as far as they extend beyond providing services to life scientists) are being called into question [3].

### The future of bioinformatic work

Yet bioinformatic work is crucial to contemporary life sciences, and the centrality of this work to new discoveries will increase. While, for now, the organisational and institutional arrangements of bioinformatics are characterised by a lack of power, as bioinformatics develops as an [inter-]discipline – matures even – it may be that the journals, departments, and courses that have sprung up over the past decade will help produce a defined field with a clear sense of its own identity. The development of disciplines is a generational process. And, with successive generations come different attitudes to what bioinformatics is; for example, our research has found that the founder generation is less likely to see bioinformatics as a distinct discipline than those who have followed [2, 12]. This should not be surprising, as the ‘forerunners’ and ‘founders’ formed the discipline in the context of a quite different arrangement of social structures than that which the ‘followers’ now inhabit.

And where do the followers go next? There are a number of trajectories that can be envisaged for the field of bioinformatics, all of which are recognisable in current research practices. One future is bioinformatics as an academic discipline, which entails departments, undergraduate and postgraduate courses, professorial chairs and the other structural elements of an established discipline. Many of these features are already in place, if unevenly distributed. Such a trajectory brings with it institutional and cultural power, esteem, and credit. However, some of the ‘founder’ generation with whom we have spoken see this future as undesirable, preferring that bioinformatics remain a tool in the repertoire of the life sciences, no more (or less) distinct than the use of any other experimental or analytic technique. Bioinformatics also has a future as a service – a set of skills supplied to the life sciences when required. In some universities this has been institutionalised as an academic service, in which bioinformaticians inhabit the same spaces as their ‘wet lab’ collaborators but occupy a different, and in many regards inferior institutional position. In other cases, this service will be ‘bought in’ through commercial channels, a situation which places bioinformaticians firmly outside the core circuits of scientific esteem and reward, regardless of their epistemic centrality, placing them in a very different ‘reward economy’.

We suspect that the future will almost certainly involve a combination of these ways of doing bioinformatics, and being a bioinformatician will mean many things, just as those working in ‘wet labs’ range from senior scientists at the core of the system of academic credit and reward, through to technicians working in the same laboratories to those with scientific training working for commercial firms that supply contract services to academic science.

## Conclusion

It is possible to look at the ‘black boxing’ of expertise – whether in tools, procedures, or contracted services – though an optimistic or pessimistic lens. Black boxing, is above else, a feature of the success of a science, method or tool. As scientific and computational work become reliable and coded into algorithms, software or machines, the process becomes less important and the focus shifts towards the inputs and outputs [13]. From a pessimistic point of view, this could mean that maintenance of the tool or service is the only role left. However, we increasingly see bioinformaticians co-designing laboratory experiments and entire studies to optimise inputs, and by consequence, optimise outputs. Bioinformaticians are, without physically producing primary inscriptions, increasingly taking responsibility for them. But despite that responsibility growing, translation of these contributions into scientific credit lags well behind.

Nevertheless, the interdisciplinary model of science is here to stay, whether labelled ‘new biology’, ‘big biology’ or otherwise [14, 15]. As evaluations of scientific practices shift towards impact, room for manoeuvring opens up for bioinformaticians. In the pursuit of relevance and impact, future scientific careers will increasingly involve playing the role of a fractional scientist. This involves combining a variety of expertises and epistemic aspirations [16], but also various roles: those of researcher, manager, service-provider, academic entrepreneur, and salesperson. If anything, through their careers in the shadows cast by the light of scientific prestige, bioinformaticians have nurtured this diverse set of skills. The biographies of tomorrow’s bioinformatic scientists will be characterised by blending, synthesis and integration, while standing firmly on the foundations of a discipline [17, 18].
